# Biomarkers of peripheral blood neutrophil extracellular traps in the diagnosis and progression of malignant tumors

**DOI:** 10.1002/cam4.6935

**Published:** 2024-01-17

**Authors:** Min Wang, Xiaoyan Lv, Ying Wang, Yao Li, Honghong Li, Zhongjun Shen, Liyan Zhao

**Affiliations:** ^1^ Department of Blood Transfusion Second Hospital of Jilin University Changchun China; ^2^ Department of Experimental Medicine Second Hospital of Jilin University Changchun China

**Keywords:** cell‐free DNA, citrullinated histone H3, neutrophil extracellular traps, systemic inflammatory response, tumor‐associated thrombosis, tumor progression

## Abstract

**Background and Aims:**

The mortality rate associated with malignant tumors remains high and there is a lack of effective diagnostic and tumor progression markers. Neutrophil extracellular traps (NETs) can promote tumor‐associated thrombosis, invasive metastasis, and inflammatory responses, but there is a lack of research on the value of measuring NETs in the peripheral blood of patients with malignancies.

**Methods:**

We included 263 patients with malignancies (55 gliomas, 101 ovarian, 64 colorectal, and 43 lung cancers) and 75 healthy controls in this study. We compared the levels of citrullinated histone H3 (citH3), cell‐free DNA (cfDNA), and systemic inflammation‐related parameters, including neutrophils, lymphocytes, monocytes, platelets, neutrophil‐to‐lymphocyte ratio, monocyte‐to‐lymphocyte ratio, platelet‐to‐lymphocyte ratio, systemic immune inflammation index, and systemic inflammation response index. We assessed the value of changes in NETs in peripheral blood to determine the diagnosis, venous thromboembolism, clinical staging, and systemic inflammatory response in patients with malignancy.

**Results:**

The levels of citH3 and cfDNA in peripheral blood can distinguish between healthy controls and tumor patients. The levels of citH3 and cfDNA before clinical intervention did not predict the risk of combined venous thromboembolism in oncology patients in the short‐term after clinical intervention. The levels of citH3, cfDNA, and systemic inflammation‐related parameters in the peripheral blood of tumor patients increased with the clinical stage. There was a correlation between cfDNA levels in peripheral blood and systemic inflammation‐related parameters in tumor patients, and this correlation was more significant in patients with advanced tumors.

**Conclusions:**

Changes in NETs in the peripheral blood differ between healthy controls and patients with malignant tumors. NETs may be involved in tumor‐induced systemic inflammatory responses through interaction with circulating inflammatory cells, thus promoting tumor progression. NETs may be used as markers to assist in the diagnosis and progression of tumor malignancy.

## 1 INTRODUCTION

The incidence and mortality rate of malignant tumors remain high. Progressive malignancies can also seriously result in significant family and socio‐economic burdens.^1^ There are usually asymptomatic manifestations in the early stages of malignant tumor development, and most patients are diagnosed in the middle or late stages, resulting in treatment difficulties. Tumor progression is the most fundamental cause of death.^2^ Tumor‐related thrombosis, organ metastasis, and associated inflammatory responses can promote tumor progression.^3^, ^4^ Thus, early detection, diagnosis, and treatment can significantly impact the prognosis of malignant tumors, increase survival, and improve quality of life.

Neutrophil extracellular traps (NETs) are an extracellular network of fibers released by neutrophils that consist of depolymerized DNA scaffolds complexed with histones, elastase (NE), and myeloperoxidase (MPO).^5^ The role of NETs in tumors was first proposed in 2012, and the presence of NETs was first confirmed in pediatric Ewing sarcoma tissue in 2013.^6^, ^7^ Studies have shown that NETs promote tumor‐associated thrombosis by providing a scaffold for the coagulation cascade and activating internal and external coagulation pathways.^8^, ^9^, ^10^ This also suggests that biomarkers of NETs formation are associated with the occurrence of blood clots in cancer patients, which may be a new pathological mechanism of tumor‐associated thrombus. Some studies have shown that NETs can promote aggressive tumor metastasis by inducing epithelial‐mesenchymal transformation of tumor cells, shielding circulating tumor cells, regulating premetastatic niches, and driving dormant cancer cells to reenter the cell cycle.^11^, ^12^, ^13^, ^14^, ^15^ NETs in the tumor microenvironment (TME) can also trigger local inflammatory responses by interacting with inflammatory cells, thereby promoting tumor progression especially in monocytes/macrophages.^16^, ^17^ Therefore, NETs play a role in thrombosis, tumor metastasis, and systemic inflammation.

NETs have been detected in primary tumors, metastatic tumor tissues, and the circulatory system of tumors.^18^, ^19^, ^20^ This may be because NETs are degraded by extracellular DNA enzymes into soluble products that are released into the peripheral blood.^21^ Studies have shown that changes in infiltrated NETs in tumor tissue correlate with changes in NETs in the peripheral blood.^22^ At present, detection of NETs in the peripheral blood is mainly through the combination of blood products related to NETs, such as citrullinated histone H3 (citH3), cell‐free DNA (cfDNA), and nucleosomes, and complexes of NETs‐related enzymes with DNA. The concentration of inflammatory cells in the peripheral circulation of tumor patients can reflect systemic inflammation related to the tumor,^23^ including neutrophils (Ne), lymphocytes (Ly), monocytes (M), platelet (PLT) counts, and their derived parameters such as neutrophil‐to‐lymphocyte ratio (NLR), monocyte‐to‐lymphocyte ratio (MLR), platelet‐to‐lymphocyte ratio (PLR), systemic immune inflammation index (SII),^24^ and systemic inflammation response index (SIRI).^25^

Although a number of studies have demonstrated that NETs are associated with tumor‐related thrombosis, invasive metastasis, and related inflammatory responses, these studies have mainly focused on cellular and animal experiments, and there is a lack of in‐depth clinical data on the value of measuring NETs in the peripheral blood as a marker of tumor progression. In addition, previous studies on NETs in malignant tumors have mainly focused on breast cancer and some digestive tract tumors, but there are fewer studies related to other systemic tumors, such as glioma, ovarian cancer, and lung cancer. In this study, we recruited patients with four different systemic malignancies (glioma, ovarian cancer, colorectal cancer, and lung cancer) to assess the value of NETs in peripheral blood in the diagnosis of tumor, venous thromboembolism (VTE), clinical stage and systemic inflammatory response in patients with malignancy.

## 2 MATERIALS AND METHODS

### 2.1 Study subjects

From June 2021 to November 2022, 263 patients diagnosed with malignant tumors in the Department of Neurosurgery, Jilin University First Hospital, and the Department of Gynecology, Gastrointestinal Surgery, and Thoracic Surgery, Jilin University Second Hospital were randomly enrolled in the tumor group in this study. In addition, 75 participants who were age‐ and sex‐matched to the tumor group and who had no abnormalities upon physical examination at the Second Hospital of Jilin University during the same period were enrolled in the control group. All tumor patients were followed for 1 month after treatment. This study was approved by the Ethics Committee of the First Hospital of Jilin University and the Second Hospital of Jilin University (19K127‐001 and 2022‐002, respectively). All participants signed informed consent.

Inclusion criteria were as follows: (1) age ≥ 18 years; (2) patients did not undergo any clinical intervention, such as surgery, chemotherapy, radiotherapy, or biological therapy; (3) pathological diagnosis of malignant tumors; (4) clinical data were relatively complete for age, gender, primary tumor site, treatment method, pathological type, degree of differentiation, lymph node metastasis, distant metastasis, clinical stage, vascular ultrasound report before and after treatment; and (5) signed the informed consent form and were willing to follow up in the trial. Exclusion criteria were as follows: (1) patient had multiple malignancies in the past or at the same time; (2) acute infectious diseases, autoimmune diseases, or uncontrolled chronic diseases, such as chronic obstructive pulmonary disease and diabetes; (3) venous or arterial thromboembolism in the past 3 months, and anticoagulant and/or antiplatelet therapy such as low molecular weight heparin, warfarin, or clopidogrel; (4) other factors that may results in study termination as judged by the investigator. The general data of the subjects are compared in Table 1.

**TABLE 1:** Comparison of general information of the study patients.

	Control	Tumor	Glioma		Ovarian cancer		Colorectal cancer		Lung cancer	
*N*	75	263	55		101		64		43	
Age	54.0 ± 8.98	56.2 ± 10.5	52.9 ± 14.8		55.5 ± 10.4		58.2 ± 7.9**		58.9 ± 6.7**	
Female n (%)	47(62.7)	163 (62.0)	22 (40.0)*		101 (100.0)**		25 (39.1)**		15 (34.9)**	
BMI(kg/m^2^)	‐	23.32 ± 3.41	22.87 ± 3.47		23.92 ± 3.66		22.92 ± 3.33		22.85 ± 2.66	
Clinical Stage^a^										
I–II	‐	121 (46.0)	17 (30.9)		47 (46.5)		33 (51.6)		24 (55.8)	
III–IV	‐	142 (54.0)	38 (69.1)		54 (53.5)		31 (48.4)		19 (44.2)	
Combined VTE^b^	‐	32 (12.2)	18 (32.7)		5 (5.0)		2 (3.1)		7 (16.3)	
Pathological type	‐	‐	Glioblastoma	25	Plasmacytoma	70	Adenocarcinoma	64	Adenocarcinoma	33
	‐	‐	Oligodendroglioma	7	Endometrioid carcinoma	11			Squamous carcinoma	9
	‐	‐	Astrocytoma	20	Clear cell carcinoma	8			Adenosquamous carcinoma	1
	‐	‐	Ventricular meningioma	3	Mucinous carcinoma	3				
	‐	‐			Non‐epithelial tumors	9				
Differentiation	‐	‐	Poor	40	Poor	78	Poor	2	Poor	16
	‐	‐	Moderate and well	15	Moderate and well	23	Moderate and well	62	Moderate and well	27
citH3(ng/mL)	173.93 ± 45.93	245.17 ± 76.57**	311.03 ± 80.45**		250.16 ± 71.31**		207.25 ± 55.52**		205.66 ± 44.66**	
cfDNA(ng/mL)	167.74 ± 20.84	262.15 ± 73.14**	285.91 ± 71.28**		240.88 ± 75.87**		279.03 ± 68.37**		256.57 ± 61.85**	
Ne(×10^9^ L^−1^)	3.20(2.57,3.90)	3.93 (3.10,5.06) **	3.49 (2.66,4.50)		4.50 (3.67,5.44)**		3.69 (3.10,4.42)**		3.83 (3.10,5.50)**	
Ly(×10^9^ L^−1^)	1.90(1.60,2.30)	1.60 (1.30,2.00) **	1.86 (1.50,2.34)		1.40 (1.20,1.70)**		1.60 (1.40,2.00)**		1.70 (1.30,2.10)**	
M(×10^9^ L^−1^)	0.30(0.30,0.40)	0.47 (0.40,0.52) **	0.49 (0.40,0.60)**		0.40 (0.30,0.50)**		0.45 (0.40,0.50)**		0.50 (0.40,0.60)**	
PLT(×10^9^ L^−1^)	238.00 (206.00,270.00)	256.00 (209.00,310.00) *	221.00 (180.00,256.00)*		287.00 (229.50,358.90)**		261.00 (207.50,309.00)*		238.00 (209.40,294.00)	
NLR	1.67(1.34,2.00)	2.45 (1.80,3.58) **	1.82 (1.51,2.42)*		3.21 (2.24,4.46)**		2.32 (1.77,3.09)**		2.56 (1.95,3.07)**	
MLR	0.17 (0.13,0.20)	0.27 (0.22,0.36)**	0.27 (0.19,0.35)**		0.29 (0.22,0.39)**		0.25 (0.24,0.33)**		0.28 (0.21,0.41)**	
PLR	125.63 (103.04,153.33)	158.33 (120.00,212.14) **	117.00 (96.73,140.95)		193.33 (145.51,282.38)**		161.90 (131.80,215.54)**		150.63 (115.00,204.76)**	
SII	404.43 (319.30,501.16)	621.50 (440.53,974.44) **	409.96 (279.63,558.33)		840.38 (554.86,1523.48)**		563.74 (467.42,809.92)**		632.54 (433.36,861.40)**	
SIRI	0.49(0.36,0.72)	1.10 (0.74,1.65) **	0.82 (0.64,1.59)**		1.25 (0.80,1.98)**		0.96 (0.76,1.33)**		1.24 (0.71,1.65)**	

*Note*: *p < 0.05 and **p < 0.01 compared with the control group.

Abbreviations: Ly, lymphocytes; M, monocytes; MLR, monocyte‐to‐lymphocyte ratio; Ne, neutrophils; NLR, neutrophil‐to‐lymphocyte ratio; PLR, platelet‐to‐lymphocyte ratio; PLT, platelets; SII, systemic immune inflammation index; SIRI, systemic inflammation response index; ‐, not covered.

^a^ Gliomas are graded according to the WHO. Considering that the transformation of low‐grade (I–II) to high‐grade (III–IV) in glioma and the transformation of early‐stage (I–II) to advanced‐stage (III–IV) in the other three tumors both indicate the progression of malignancy, the grading of glioma and the staging of the other three tumors have been unified here as “clinical stage”.

^b^ Refers to the combination of VTE in a short period of time after clinical intervention in patients with malignancy.

### 2.2 Collection and processing of samples

Blood samples were collected from the tumor group and the control group via venipuncture before clinical intervention and on the day of physical examination. Venous blood was collected in vacuum blood collection tubes containing silica gel particles and separation gel. After collection, the tubes were kept at room temperature until complete coagulation, then centrifuged at 1000 × ***g*** for 20 min in a low‐temperature centrifuge after 2–4 h. Finally, blood serum was stored in separate packaging at −80°C for the detection of citH3 and cfDNA. In addition, another 2 mL of EDTA anticoagulated blood was taken from the tumor and control groups for blood examination. Neutrophil, lymphocyte, monocyte, and platelet counts were measured using the fully automated blood cell analyzer Sysmex‐XN2000 (Sysmex, Japan) with matched fluorescent staining solution, hemolytic products, and diluents. NLR = N/L, MLR = M/L, PLR = P/L, SII = P × N/L, SIRI = M × N/L. N, M, L, and P represent neutrophil, monocyte, lymphocyte, and platelet counts in peripheral blood, respectively.

### 2.3 Culture of glioma cells and preparation of conditioned medium (CM)

The human glioma cell line LN18 (American Type Culture Collection, USA) was cultured in DMEM medium (Gibco, USA) containing 5% fetal bovine serum and 1% penicillin–streptomycin. CM was harvested from the tumor cells as previously described.^9^, ^14^, ^26^ LN18 cells were cultured in 6‐well plates until the cell density reached 80%, and then washed twice with PBS and replaced with serum‐free RPMI‐1640 medium (Procell, China) and incubated at 37°C, 5% CO_2_ for 24 h. After 24 h, the media was collected and centrifuged at 4000 rpm for 5 min at 4°C. The CM was then filtered through a 0.22 μm filter and stored at −80°C. Non‐conditioned RPMI‐1640 culture medium without serum was used as a negative control for in vitro experiments.

### 2.4 Isolation of neutrophils and induction of NETs in vitro

We collected 5 mL whole blood of healthy volunteers, anticoagulated the samples with EDTA, spread the blood on 5 mL PolymorPrep separation solution (Axis‐Shield, Norway), and centrifuged at 500 × ***g*** for 30 min at room temperature. After centrifugation, the layer containing neutrophils was collected and washed twice with PBS. The red blood cells were lysed with erythrocyte lysate (TBD, China) and the purified neutrophils were resuspended in RPMI‐1640 medium without serum. Neutrophils were identified as having >95% purity using Wright's Giemsa complex staining and > 98% viability using Trypan blue staining (Figure S1). The freshly isolated neutrophils were inoculated on polylysine‐coated slides (WHB Scientific, China) in 24‐well plates (3 × 10^5^ cells/well) and incubated at 37°C for 40 min in a 5% CO_2_ incubator. Subsequently, PMA (25 nM, MCE, USA) was added and incubated for 1–4 h. The CM from the glioma cells was considered the tumor group, and the non‐CM was considered the control group, both incubated for 4 h.

### 2.5 Fluorescent staining of NETs

The slides were fixed with 4% paraformaldehyde for 15 min and washed three times with PBS. The cells were stained with Sytox Green (1 μM, Thermo Fisher Scientific, USA) and Hoechst 33342 (1:100, Beyotime, China) for 10 min protected from light, washed three times with PBS, and quickly photographed with an inverted fluorescence microscope (Olympus, Japan). The area of NETs was quantified using ImageJ with the parameter “average NETs area per cell”.

### 2.6 Immunofluorescence staining of NETs

The slides were fixed with 4% paraformaldehyde for 15 min and washed three times with PBS. The cells were permeabilized with 0.1% Triton X‐100 for 5 min and washed three times with PBS. The slides were then blocked with PBS containing 1% BSA, 10% normal goat serum, and 0.3 M glycine for 30 min before incubation with anti‐citH3 antibody ab5103 (1:1000, Abcam, UK) at 4°C overnight. The slides were brought to room temperature, washed three times with PBS, incubated with Alexa Fluor 488 goat anti‐rabbit IgG (H + L) (1:400, Yeasen, China) at room temperature in the dark for 1 h, and then washed three times with PBS. Finally, the slides were sealed with an anti‐fluorescent quenching solution (Beyotime, China) containing DAPI at room temperature for 5 min in the dark. Images were acquired as soon as possible under a fluorescence microscope (Olympus, Japan).

### 2.7 Detection of citH3 and cfDNA

citH3 was detected in the serum using the human citrullinated histone H3 Elisa kit (Huiyin Biotechnology, China) according to the manufacturer's instructions. Optical density (OD) was measured at 450 nm using an enzyme reader (Bio Tek, USA). The actual concentrations of citH3 in the samples were calculated according to the OD value of the standard curve. To quantitatively measure cfDNA in serum using fluorescent nucleic acid staining, the Quant‐iT PicoGreen dsDNA kit (Thermo Fisher Scientific, USA) was diluted 1:200 in TE buffer (1×) to prepare an aqueous solution. In brief, 50 μL of the test serum was added to a 96‐well plate, followed by 50 μL of TE buffer, and the sample was diluted twofold with TE buffer. Finally, 100 μL of Quant‐it PicoGreen dsDNA aqueous solution was added, and the plate was incubated in the dark at room temperature for 5 min. The fluorescence intensity was measured at an excitation wavelength of 480 nm and an emission wavelength of 520 nm using a multifunctional microplate detector (Bio Tek, USA). The actual concentration of cfDNA in the sample was calculated based on the fluorescence intensity of the standard curve.

### 2.8 Statistical analysis

SPSS 25.0, ImageJ, and GraphPad Prism 9.0 software were used for statistical analysis and graphing. All variables were tested for normality using the Shapiro–Wilk method. Variables that met normal distribution are expressed as^−^x ± s; the *t*‐test was used for comparisons between two groups, and GraphPad was used to draw a column chart. Variables that did not meet normal distribution are represented as M (P_25_, P_75_); the Mann–Whitney *U*‐test was used for comparisons between two groups, and GraphPad Prism was used to draw box plots. Count data are expressed as frequency (rate); the chi‐squared test was used for comparisons between the two groups. The Spearman correlation coefficient was used for correlation analysis between variables. Receiver operating characteristic (ROC) curves and area under the curve (AUC) were calculated for all continuous variables of interest, and the Youden index was used to maximize the difference between sensitivity and specificity to individualize the best cut‐off value for each parameter. All cell experiments were repeated three times.

Four types of tumors were included in this study. Judging criterion were developed considering the variability between the tumor group and each type of tumor, and between different types of tumors: each index was statistically significant in patients in the tumor group, two and more types of tumor groups at the same time, or only in patients in two and more types of tumor groups, and we considered it if it met these two conditions had clinical significance. *p* < 0.05 was considered statistically significant.

## 3 RESULTS

### 3.1 PMA and glioma cells stimulated neutrophils to release NETs

**FIGURE 1:**
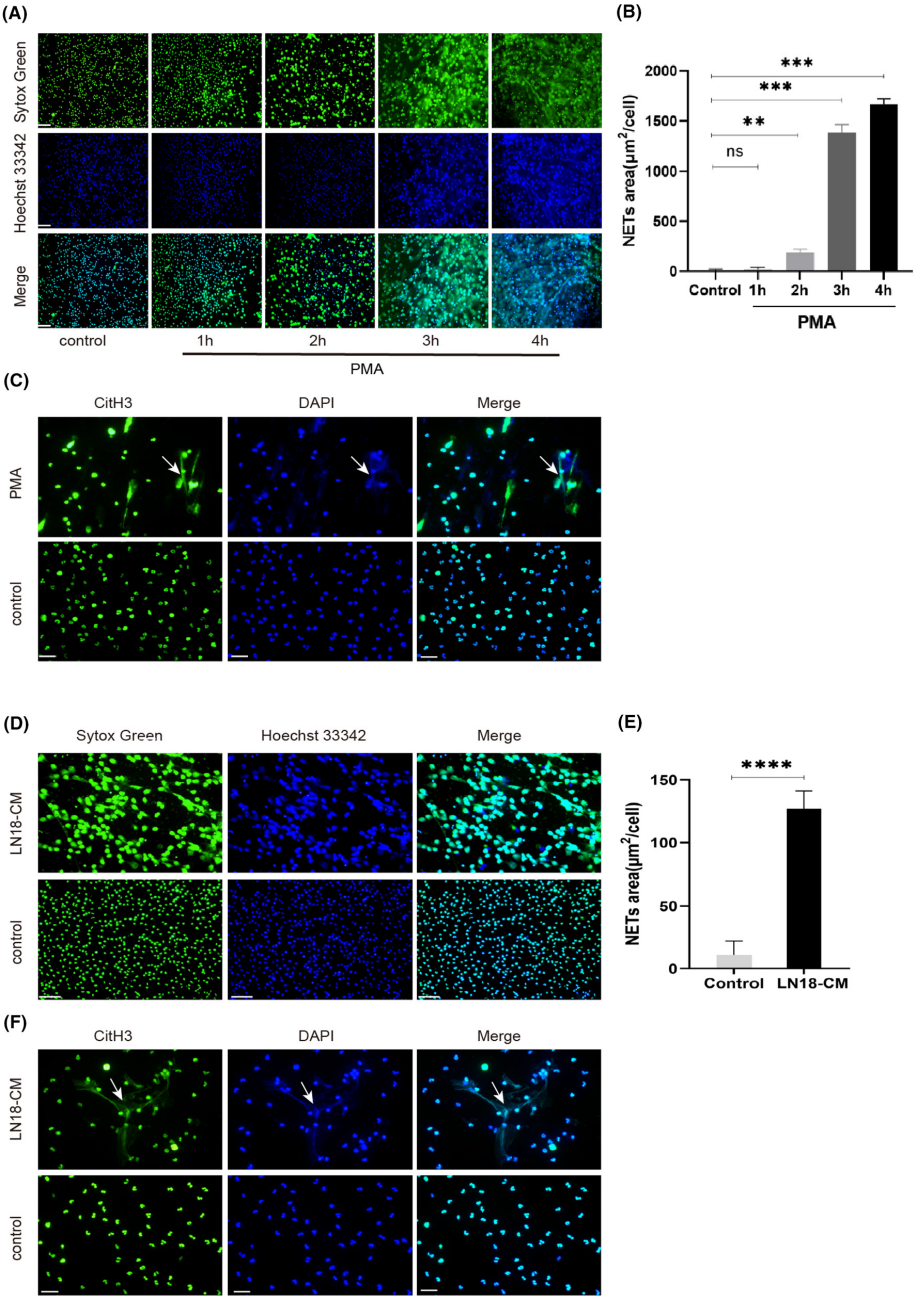
PMA and glioma cells stimulated neutrophils to release NETs. (A) Neutrophils were stimulated with PMA for 1, 2, 3, and 4 h, respectively, and then stained with Sytox Green (green) and Hoechst 33342 (blue) to observe NETs (scale: 50 μm). (B) Quantitative results of fluorescent staining of the area of PMA‐stimulated neutrophils releasing NETs at different time points (ns = not statistically significant, **p < 0.01, ***p < 0.001). (C) PMA stimulation of neutrophils for 4 h, and immunofluorescence staining of both citH3 (green) and DNA (DAPI, blue) was performed to visualize NETs, the white arrows indicate NETs (scale bar: 20 μm). (D) Fluorescence‐stained images of LN18 CM stimulated neutrophils for 4 h to release NETs (scale bar: 50 μm). (E) Quantitative results of fluorescence staining of the area of LN18 CM‐stimulated neutrophils releasing NETs at 4 h (****p < 0.0001). (F) Immunofluorescence staining images of LN18 CM‐stimulated neutrophils releasing NETs at 4 h, the white arrows indicate NETs (scale bar: 20 μm). Error bars in B and E represent SD.

Following PMA stimulation of neutrophils in vitro, fluorescence staining showed that most neutrophils released NETs, and large fibrous substances cross‐linked together to form a typical fibrous network structure. In the control group, only a small proportion of cells lost the shape of the lobular nucleus, and the nucleus became swollen at 4 h (Figure 1A). The quantitative results indicated that the released NETs area was significantly larger than that of the control group over time (Figure 1B). Furthermore, immunofluorescence staining of both citH3 and DNA resulted in a similar observation of fibrillar NETs production (Figure 1C). This demonstrates that citH3 and DNA are indeed components of NETs.

After coculturing CM of the glioma cell line LN18 with neutrophils for 4 h, fluorescent staining and quantification showed that neutrophils clearly produced an extracellular fibrous substance similar to PMA stimulation, namely NETs (Figure 1D,E). Immunofluorescence staining of both citH3 and DNA further confirmed the production of NETs (Figure 1F). These results indicate the association between malignancy and the production of NETs.

### 3.2 Comparison of NETs in the peripheral blood

**FIGURE 2:**
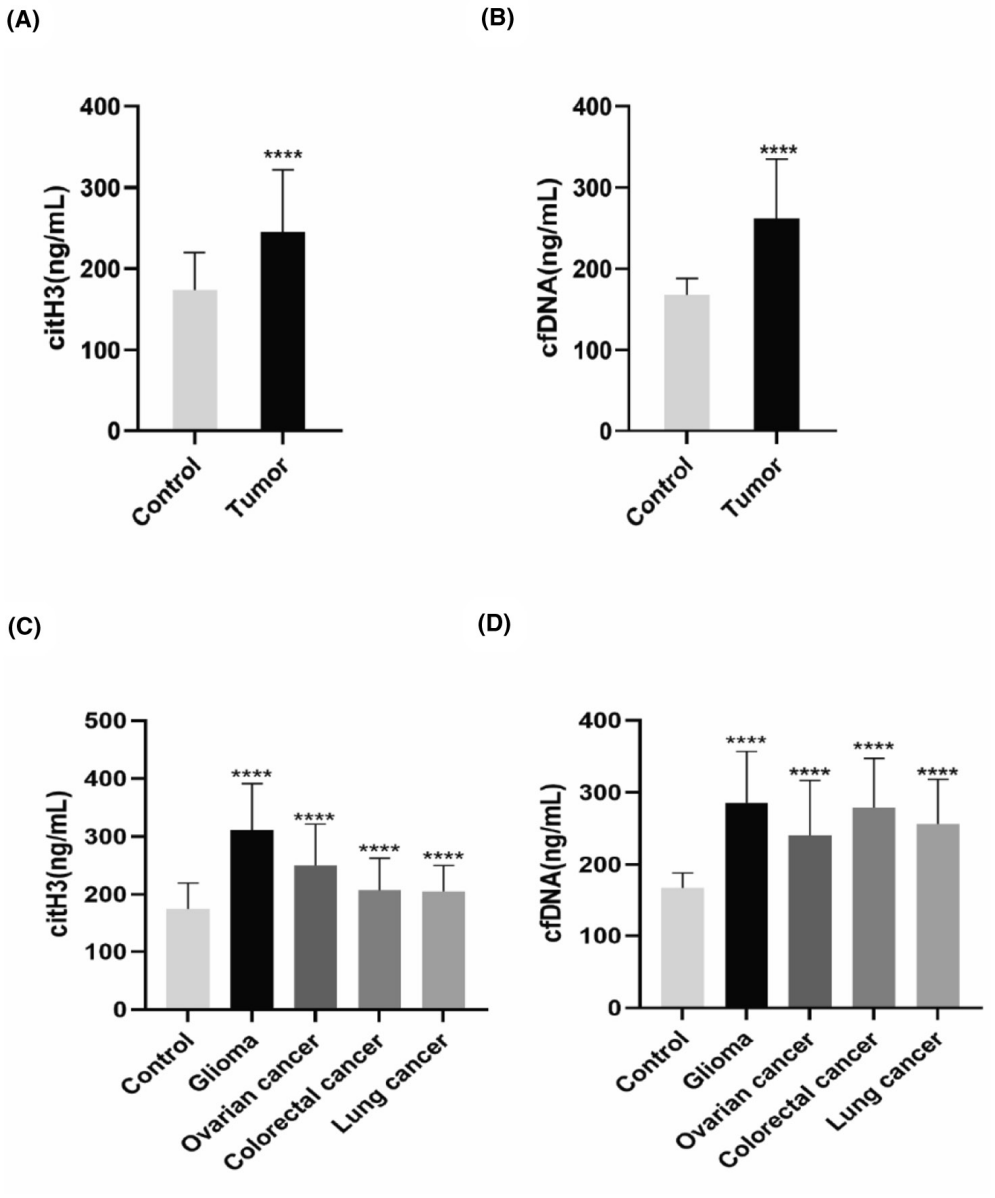
Comparison of the levels of citH3 and cfDNA in the peripheral blood of the study patients. (A) The levels of citH3 in the peripheral blood of patients in the tumor group were higher than those in the control group. (B) The levels of cfDNA in the peripheral blood of patients in the tumor group were higher than those in the control group. (C) The levels of citH3 in the peripheral blood of patients in each type of tumor group were higher than those in the control group. (D) The levels of cfDNA in the peripheral blood of patients in each type of tumor group were higher than those in the control group. *t*‐test; ****p < 0.0001. Error bars in A–D represent SD.

Compared with the control group, the levels of citH3 and cfDNA in the peripheral blood were elevated in the tumor group and in each type of tumor (glioma, ovarian cancer, colorectal cancer, and lung cancer groups) (all *p* < 0.0001, Figure 2).

### 3.3 Diagnostic value of NETs in the peripheral blood

**FIGURE 3:**
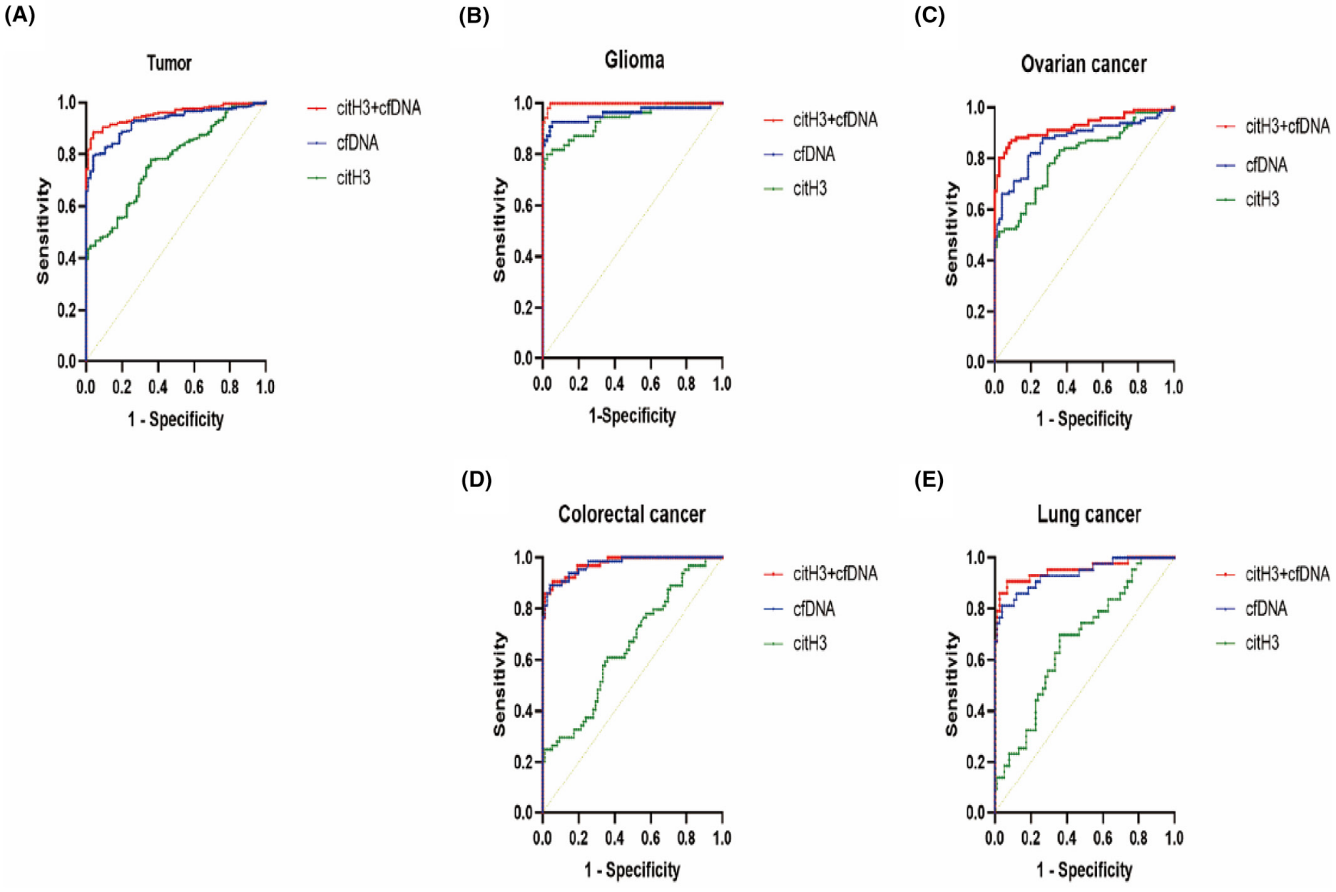
ROC curves of citH3, cfDNA, and citH3 + cfDNA in the tumor group and in each type of tumor. (A) The diagnostic capacities of citH3, cfDNA and citH3+cfDNA were sequentially higher in the tumor group (AUC 0.775, 0.925 and 0.956, respectively); (B) The diagnostic capacities of citH3, cfDNA and citH3+cfDNA were sequentially higher in the glioma group (AUC 0.936, 0.959 and 0.998, respectively); (C) The diagnostic capacities of citH3, cfDNA and citH3+cfDNA were sequentially higher in the ovarian cancer group (AUC of 0.807, 0.871 and 0.927, respectively); (D) The diagnostic capacities of citH3, cfDNA and citH3+cfDNA were sequentially higher in the colorectal cancer group (AUC of 0.654, 0.973 and 0.976, respectively); (E) The diagnostic capacities of citH3, cfDNA and citH3+cfDNA were sequentially higher in the lung cancer group (AUC of 0.672, 0.937 and 0.953, respectively).

The ROC curves results showed that the AUC of citH3, cfDNA, and citH3 + cfDNA increased sequentially in the tumor group and various types of tumors (Figure 3). citH3 exhibited higher specificity than cfDNA in the tumor group, as well as for glioma and ovarian cancer. The sensitivity of cfDNA was consistently higher than that of citH3 in the tumor group and each type of tumor. The combination of citH3 + cfDNA significantly increased sensitivity and slightly decreased specificity when compared with either citH3 or cfDNA alone (Table 2). These results combined with the AUC analysis showed that the combination of citH3 + cfDNA could more accurately distinguish between the control and tumor groups, as well as each type of tumor.

**TABLE 2:** Diagnostic performance of citH3, cfDNA, and citH3 + cfDNA in the tumor group and in each type of tumor.

	AUC (95% CI)	Sensitivity	Specificity	Youden Index	Cut‐off
Control‐Tumor					
citH3	0.775 (0.722–0.828)	0.437	0.987	0.424	248.41
cfDNA	0.925 (0.898–0.953)	0.795	0.960	0.755	201.40
citH3 + cfDNA					
	0.956 (0.936–0.976)	0.886	0.960	0.846	‐
Control‐Glioma					
citH3	0.936 (0.893–0.980)	0.800	0.973	0.773	244.69
cfDNA	0.959 (0.918–1.000)	0.927	0.947	0.874	200.78
citH3 + cfDNA					
	0.998 (0.995–1.000)	1.000	0.960	0.960	‐
Control‐Ovarian cancer					
citH3	0.807 (0.745–0.870)	0.515	0.973	0.488	244.67
cfDNA	0.871 (0.818,0.924)	0.812	0.813	0.625	183.36
citH3 + cfDNA	0.927 (0.888–0.967)	0.861	0.920	0.781	‐
Control‐Colorectal cancer					
citH3	0.654 (0.563–0.744)	0.609	0.640	0.249	187.32
cfDNA	0.973 (0.951–0.995)	0.891	0.960	0.851	203.51
citH3 + cfDNA	0.976 (0.956–0.996)	0.906	0.947	0.853	‐
Control‐Lung cancer					
citH3	0.672 (0.573–0.771)	0.698	0.640	0.338	187.05
cfDNA	0.937 (0.889–0.985)	0.814	0.960	0.774	204.36
citH3 + cfDNA	0.953 (0.910–0.997)	0.907	0.933	0.840	‐

### 3.4 Predictive value of NETs in the peripheral blood for determining the risk of thrombosis in tumor patients

**FIGURE 4:**
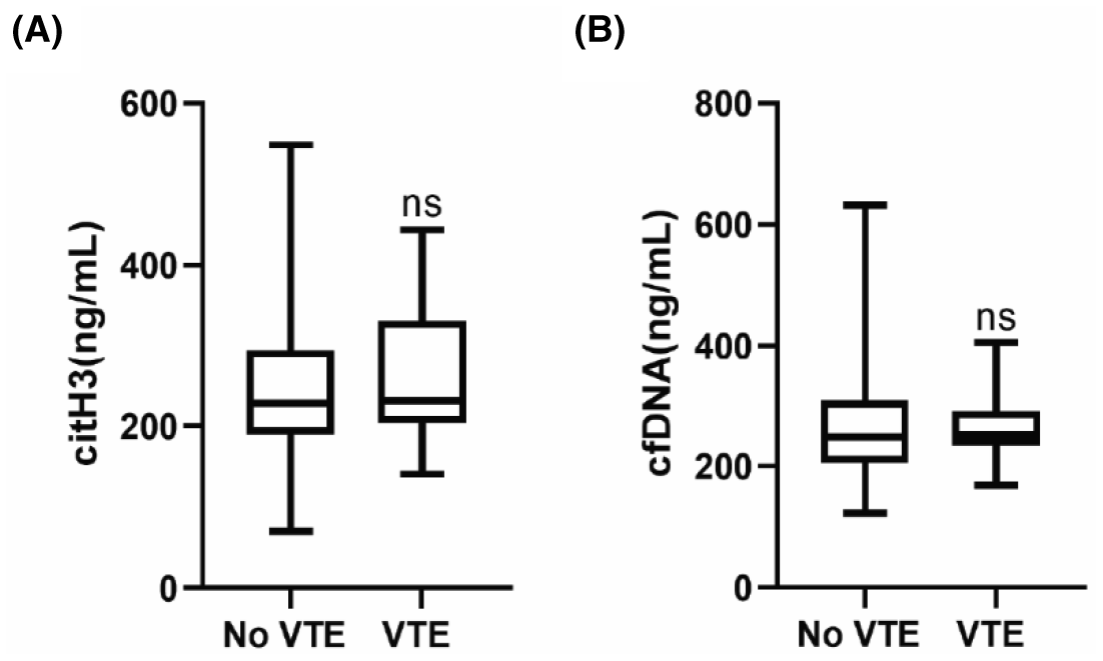
citH3 and cfDNA levels. The levels of peripheral blood citH3 (A) and cfDNA (B) before clinical intervention were compared between patients in the uncombined VTE group and the combined VTE group in the short term after the clinical intervention. Mann–Whitney U test, ns = not statistically significant. Boxes extend from the 25th to 75th percentiles, and the horizontal lines in the boxes represent the medians.

In this study, some tumor patients developed VTE during the 1‐month follow‐up period. To investigate whether levels of NETs in the peripheral blood before clinical intervention in tumor patients can predict the risk of VTE in tumor patients shortly after clinical intervention, we compared citH3 and cfDNA levels between patients in the uncombined VTE group and the combined VTE group. The results showed that there was no statistically significant difference in citH3 and cfDNA levels between the two groups (Figure 4).

### 3.5 Relationship between changes in NETs in the peripheral blood and the clinical stage of tumors

**FIGURE 5:**
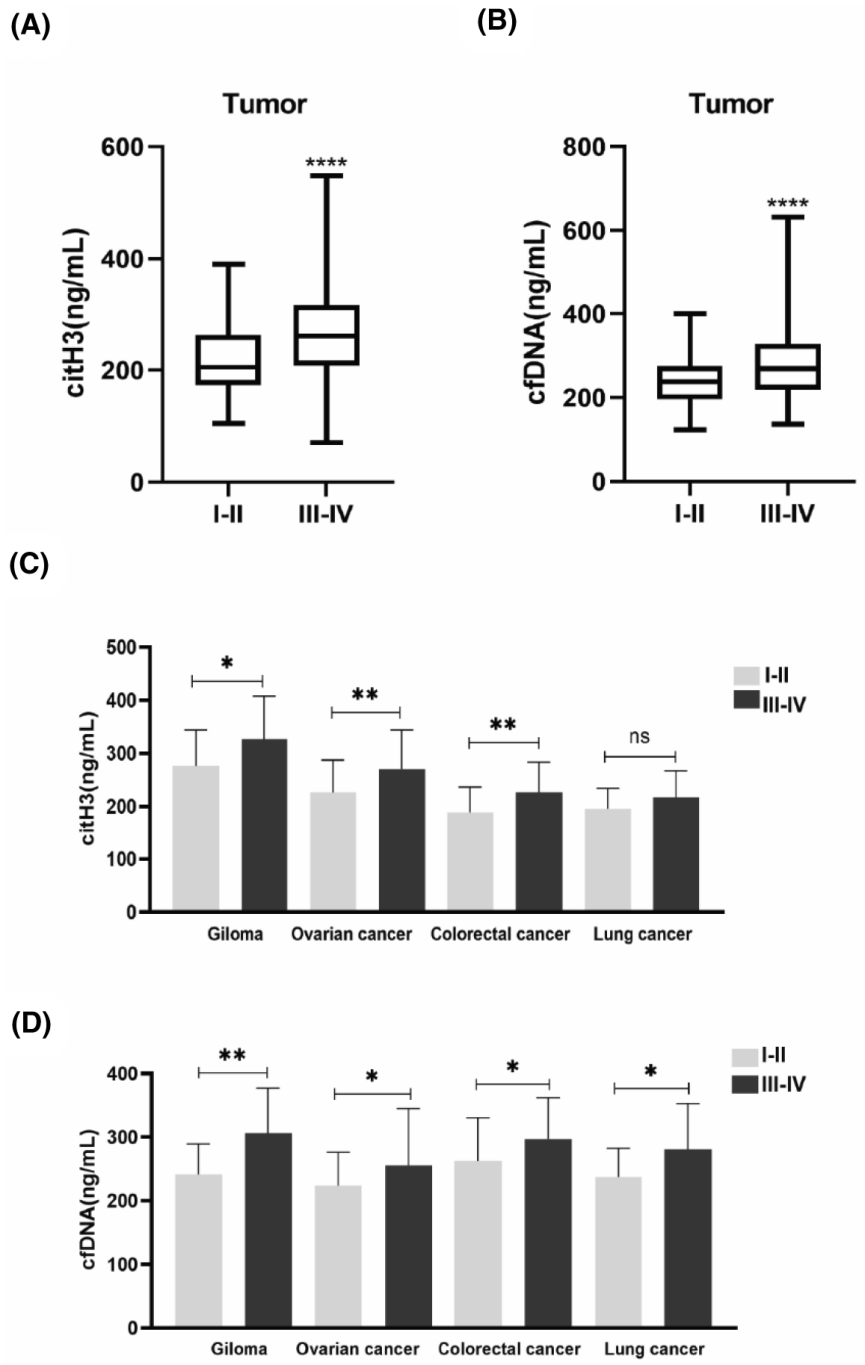
Elevated levels of citH3 and cfDNA in the peripheral blood of tumor patients correlates with tumor clinical stage. (A) The levels of citH3 in the peripheral blood of patients in the advanced tumor group were higher than those of patients in the early tumor group. (B) The levels of cfDNA in the peripheral blood of patients in the advanced tumor group were higher than those of patients in the early tumor group. Boxes extend from the 25th to 75th percentiles, and the horizontal lines in the boxes represent the medians. (C) In each type of tumor group, except for citH3 in the lung cancer group, the levels of citH3 in other tumors were higher in patients with advanced stage than in patients with early stage. (D) In each type of tumor group, the levels of cfDNA in patients with advanced stage were higher than in patients with early stage. (A, B) shows Mann–Whitney *U*‐test analyses; (C, D) show *t*‐test analyses; ns = not statistically significant, *p < 0.05, **p < 0.01, ****p < 0.0001. Error bars in C and D represent SD.

The transformation of low‐grade (I–II) to high‐grade (III–IV) gliomas and the early‐stage (I–II) to advanced‐stage (II–IV) transformation of the other three types of tumors both indicate the progression of malignant tumors, the grading of gliomas and the staging of the other three types of tumors are collectively referred to as “clinical stage” in our analysis. The tumor group patients were divided into early and advanced stages according to clinical stage, and the results showed that the levels of citH3 and cfDNA in advanced‐stage patients were higher than that in early‐stage patients (all *p* < 0.0001, Figure 5A,B). Patients with different types of tumors were also grouped according to clinical staging, and the results showed that except for citH3 in the lung cancer group, the levels of citH3 and cfDNA in advanced‐stage patients were higher than those in early‐stage patients in other tumors (*p* < 0.05 or *p* < 0.01, Figure 5C,D). These results suggest that the level of NETs in the peripheral blood of tumor patients increases with increases in clinical stage.

### 3.6 Correlation analysis between NETs in the peripheral blood of tumor patients and systemic inflammation‐related parameters

Table 1 shows that Ne, M, PLT, NLR, MLR, PLR, SII, and SIRI were elevated (*p* < 0.05 or *p* < 0.01) and Ly was decreased (*p* < 0.01) in patients in the tumor group, as well as in those with two and more types of tumors compared with the control group, indicating that the tumor patients were indeed in a state of chronic inflammation. The correlation between peripheral blood NETs and systemic inflammation‐related parameters in tumor patients showed that cfDNA in the peripheral blood of tumor patients was positively correlated with Ne, M, NLR, MLR, SII, and SIRI (*p* < 0.05 or *p* < 0.01, Table 3) and negatively correlated with Ly (*p* < 0.05 or *p* < 0.01, Table 3), suggesting that NETs are associated with tumor‐induced systemic inflammatory responses and that the systemic inflammatory response was mainly dominated by neutrophils and monocytes. The correlation between cfDNA in the peripheral blood and Ne, M, NLR, MLR, SII, and SIRI (*p* < 0.05 or *p* < 0.01, Table 4) in tumor patients was more significant in patients with advanced tumors. Patients in the tumor group and each type of tumor group were grouped according to clinical stage, and the results showed that NLR, MLR, SII, and SIRI (*p* < 0.05 or *p* < 0.01, Table 5) increased with increasing clinical stage in the tumor group and two types of tumor groups, and PLR (*p* < 0.05 or *p* < 0.01, Table 5) increased with increasing clinical stage in two types of tumor groups, suggesting that elevated levels of systemic inflammation‐related parameters NLR, MLR, PLR, SII, and SIRI correlate with disease progression in tumor patients.

**TABLE 3:** Correlation analysis between citH3, cfDNA, and systemic inflammation‐related parameters in peripheral blood of patients in the tumor group and each type of tumor group.

	*n*			Ne	Ly	M	PLT	NLR	MLR	PLR	SII	SIRI
Tumor	263	citH3	*r*	0.033	0.014	0.029	−0.068	0.012	0.000	−0.087	−0.015	0.025
		cfDNA	*r*	0.172 ^**^	−0.009	0.270 ^**^	0.027	0.106	0.233 ^**^	0.028	0.097	0.243 ^**^
Glioma	55	citH3	*r*	0.123	0.122	0.056	0.122	0.025	−0.058	−0.054	0.055	0.064
		cfDNA	*r*	0.534 ^**^	0.165	0.293 ^*^	0.147	0.246	0.171	0.067	0.303 ^*^	0.418 ^**^
Ovarian cancer	101	citH3	*r*	0.029	−0.115	−0.054	0.003	0.084	−0.010	0.047	0.061	0.023
		cfDNA	*r*	0.331 ^**^	−0.245 ^*^	0.331 ^**^	0.180	0.372 ^**^	0.372 ^**^	0.258 ^**^	0.339 ^**^	0.424 ^**^
Colorectal cancer	64	citH3	*r*	0.054	0.056	−0.025	−0.030	0.043	−0.040	−0.001	0.083	0.014
		cfDNA	*r*	0.043	0.027	0.143	0.130	−0.007	0.120	0.119	0.059	0.100
Lung cancer	43	citH3	*r*	−0.038	−0.131	−0.027	−0.114	0.113	0.114	0.016	0.042	0.030
		cfDNA	*r*	0.179	−0.302 ^*^	0.102	−0.007	0.322 ^*^	0.357 ^*^	0.292	0.270	0.359 ^*^

*Note*: Statistically significant results are underlined. *r* is the spearman correlation coefficient; *p < 0.05, **p < 0.01.

**TABLE 4:** Correlation analysis between citH3 and cfDNA in peripheral blood and systemic inflammation‐related parameters in patients with different stages of tumor and each type of tumor group.

	n			Ne	Ly	M	PLT	NLR	MLR	PLR	SII	SIRI
Tumor												
I–II	121	citH3	*r*	−0.011	0.044	0.027	−0.029	−0.038	−0.064	−0.093	−0.064	−0.037
		cfDNA	*r*	0.119	0.067	0.132	0.029	−0.013	0.098	−0.021	0.022	0.157
III–IV	142	citH3	*r*	−0.040	0.061	−0.049	−0.156	−0.071	−0.087	−0.171 ^*^	−0.109	−0.074
		cfDNA	*r*	0.160	−0.032	0.333 ^**^	−0.001	0.138	0.270 ^**^	0.020	0.101	0.258 ^**^
Glioma												
I–II	17	citH3	*r*	−0.431	0.059	0.010	0.238	−0.289	−0.015	−0.059	−0.230	−0.162
		cfDNA	*r*	0.480	0.108	0.107	0.096	0.159	−0.005	−0.005	0.277	0.189
III–IV	38	citH3	*r*	0.159	0.219	0.132	0.198	0.024	−0.088	−0.063	0.070	0.045
		cfDNA	*r*	0.444 ^**^	0.267	0.436 ^**^	0.287	0.174	0.238	0.097	0.281	0.441 ^**^
Ovarian cancer												
I–II	47	citH3	*r*	0.140	−0.070	−0.046	−0.068	0.102	−0.107	−0.016	0.054	0.019
		cfDNA	*r*	0.304 ^*^	−0.166	0.168	0.070	0.339 ^*^	0.249	0.179	0.278	0.410 ^**^
III–IV	54	citH3	*r*	−0.124	−0.018	−0.136	−0.142	−0.075	−0.057	−0.060	−0.111	−0.113
		cfDNA	*r*	0.343 ^*^	−0.255	0.422 ^**^	0.174	0.361 ^**^	0.410 ^**^	0.237	0.333 ^*^	0.426 ^**^
Colorectal cancer												
I–II	33	citH3	*r*	0.172	0.240	0.160	0.078	0.053	−0.012	−0.134	0.063	0.048
		cfDNA	*r*	−0.142	0.118	−0.016	0.041	−0.279	−0.175	−0.141	−0.252	−0.177
III–IV	31	citH3	*r*	−0.125	−0.183	−0.270	−0.154	−0.003	−0.054	0.145	0.047	−0.111
		cfDNA	*r*	0.187	−0.062	0.288	0.229	0.238	0.378 ^*^	0.364 ^*^	0.330	0.298
Lung cancer												
I–II	24	citH3	*r*	−0.192	−0.068	−0.352	−0.131	−0.050	−0.318	−0.102	−0.230	−0.332
		cfDNA	*r*	0.242	0.171	0.237	−0.083	0.109	0.155	−0.098	0.061	0.289
III–IV	19	citH3	*r*	−0.022	−0.037	0.207	−0.212	0.030	0.249	−0.123	−0.091	0.079
		cfDNA	*r*	0.169	−0.626 ^**^	−0.207	0.046	0.475 ^*^	0.489 ^*^	0.630 ^**^	0.474 ^*^	0.396

*Note*: Statistically significant results are underlined. *r* is the spearman correlation coefficient; *p < 0.05, **p < 0.01.

**TABLE 5:** Comparison of systemic inflammation‐related parameters in patients with different stages of tumor and each type of tumor group.

	Tumor		Glioma		Ovarian cancer		Colorectal cancer		Lung cancer	
	I–II	III–IV	I–II	III–IV	I–II	III–IV	I–II	III–IV	I–II	III–IV
*n*	121	142	17	38	47	54	33	31	24	19
Ne (×10^9^ L^−1^)	3.75 (2.90,4.70)	4.07 (3.43,5.53)_**_	3.15 (2.39,3.84)	3.70 (2.98,4.83)_*_	4.36 (3.50,5.40)	4.66 (3.94,5.62)	3.46 (2.82,4.23)	3.77 (3.30,5.10)	3.58 (3.02,4.48)	4.68 (3.14,5.90)
Ly (×10^9^ L^−1^)	1.60 (1.40,2.10)	1.60 (1.20,2.00)	2.11 (1.49,2.47)	1.81 (1.50,2.25)	1.40 (1.30,1.80)	1.40 (1.00,1.70)	1.60 (1.40,2.00)	1.60 (1.40,2.0)	1.7 (1.50,2.20)	1.50 (1.20,2.00)
M (×10^9^ L^−1^)	0.40 (0.30,0.50)	0.50 (0.40,0.60)_**_	0.49 (0.43,0.63)	0.48 (0.38,0.60)	0.40 (0.30,0.50)	0.50 (0.30,0.60)	0.40 (0.30,0.50)	0.50 (0.40,0.50)	0.40 (0.30,0.50)	0.50 (0.50,0.70)_**_
PLT (×10^9^ L^−1^)	247.30 (209.80,296.60)	262.50 (207.00,332.25)	234.00 (195.5255.5)	206.00 (197.00,256.00)	258.50 (217.40,304.80)	314.00 (261.00,430.60) **	258.00 (203.50,311.50)	264.00 (209.00,306.00)	239.50 (206.28,281.00)	233.00 (209.40,303.00)
NLR	2.33 (1.65,3.06)	2.59 (1.94,3.96)_**_	1.62 (1.18,2.20)	1.95 (1.55,2.86)	2.82 (2.08,3.59)	3.66 (2.32,5.37)_*_	2.21 (1.59,2.80)	2.44 (2.00,3.55)	2.20 (1.81,2.78)	2.75 (2.18,4.35)_**_
MLR	0.25 (0.21,0.34)	0.29 (0.23,0.40)**	0.24 (0.20,0.37)	0.27 (0.19,0.35)	0.25 (0.21,0.36)	0.30 (0.23,0.42)_*_	0.25 (0.24,0.33)	0.26 (0.23,0.40)	0.23(0.20,0.28)	0.40 (0.29,0.54)_**_
PLR	150.63 (109.71,201.54)	161.90 (124.81,224.03)	102.87 (87.50,159.46)	122.51 (97.15,139.73)	176.00 (133.04,217.00)	206.58 (166.86,388.13) **	156.58 (117.78,214.40)	161.92 (135.33,221.11)	143.54 (97.94,169.06)	196.45 (115.79,226.56)_*_
SII	533.62 (429.00,804.05)	719.77 (456.40,1165.15) **	404.26 (257.04,500.88)	410.98 (285.29,727.74)	705.83 (466.64,1174.78)	1036.48 (648.13,2114.92) **	526.68 (448.21,735.23)	621.50 (466.50,899.92)	475.50 (364.66,652.75)	779.08 (640.20,1126.19) **
SIRI	0.92 (0.70,1.39)	1.29 (0.80,1.95)_**_	0.79 (0.59,1.27)	0.86 (0.64,1.68)	1.00 (0.73,1.75)	1.51 (0.89,2.68)_*_	0.93 (0.68,1.25)	0.98 (0.81,1.60)	0.78 (0.62,1.25)	1.6 (1.29,2.18)_**_

*Note*: Statistically significant results are underlined. *p < 0.05 and **p < 0.01 compared with the early (I–II) stage.

## 4 DISCUSSION

Since their discovery in 2004, the roles of NETs in pathological processes have been continuously revealed. In recent years, NETs have been found to exist in the microenvironment of a variety of tumors, and it has been shown that NETs promote the spread and metastasis of tumor cells by capturing tumor cells in the blood or by degrading the extracellular matrix of tumor tissue by proteases on their DNA backbone.^27^, ^28^ The three main factors that affect the release of NETs from neutrophils in malignant tumors are tumor cells themselves, stromal cells in the TME, and metabolic factors.^29^ Tumor cells can induce the release of NETs by secreting stimulating factors such as ELR + CXCL chemokines,^13^ granulocyte colony‐stimulating factor,^30^ and exosomes.^31^ In tumors such as pancreatic cancer,^9^ ovarian cancer,^14^ and breast cancer,^26^ CM of tumor cells was found to stimulate the release of NETs. The process by which NETs are released is called “NETosis”.^32^ In this study, neutrophils were stimulated with PMA, and over time, fluorescence staining showed that the lobed nucleus shape of the neutrophils disappeared, the nucleus became sparse and swollen, and fibrous NETs were subsequently released and intertwined to form an extracellular reticule‐fibrillary structure. We showed that NETosis is a dynamic and continuous process, consistent with previous research.^33^ In this study, the CM of the glioma cell line LN18 was cocultured with neutrophils for 4 h, and the production of original fiber NETs was observed using fluorescence and immunofluorescence staining. Our results indicate that tumor cells do indeed participate in the release of NETs, providing strong evidence for measuring citH3 and cfDNA levels in the peripheral blood of tumor patients as a marker of NETs in subsequent experiments.

At present, there are several tumors that can be identified reliably with specific tumor markers, which has assisted in tumor screening, diagnosis, prognosis, and recurrence detection. However, there is a significant gap in research on NETs as tumor diagnostic markers. NE‐DNA complexes in gastric cancer and citH3 and cfDNA in endometrial cancer have been discovered as noninvasive markers for tumor diagnosis.^22^, ^34^, ^35^, ^36^ In this study, we found that citH3 and cfDNA levels in the peripheral blood of tumor patients with different types of tumors were significantly higher than those in the control group. Furthermore, the AUC and Youden index of citH3, cfDNA, and citH3 + cfDNA increased sequentially in the tumor group and in those with different types of tumors. The combined diagnosis of citH3 + cfDNA can maximally and authentically distinguish tumor patients, indicating the value of NETs in tumor diagnosis. However, this study also has certain limitations. We only analyzed the ROC curves of the control and tumor patients. In the future, some patients with benign tumors can be appropriately included to better judge the diagnostic value of NETs for identifying or predicting malignant tumors.

Tumors associated with VTE are the second leading cause of death. In this study, we found that citH3 and cfDNA levels in tumor patients before clinical intervention cannot predict the short‐term risk of VTE after intervention, which differs from the results of previous studies. From a clinical perspective, there may be several reasons for this: (i) All tumor patients in this study who developed VTE were in postoperative stages. The duration of surgery, presence of intra‐operative blood transfusion, and postoperative bed rest time are all risk factors for VTE. Therefore, it can be hypothesized that the reason we did not observe any significant difference in NETs between the two groups in this study is that NETs are not an independent or primary risk factor for VTE formation. (ii) The level of NETs may be dynamic. In a mouse model of colon cancer liver metastasis, hepatic ischemia–reperfusion induced an increase in NETs production caused by surgical stress.^17^ Zhang et al.^22^ also reported that the level of NETs in patients was significantly lower 1 month after gastric cancer surgery compared to before surgery. VTE incidence in this study mostly occurred within 1 week after surgery. Therefore, tumor patients may produce a large number of NETs during the postoperative stress period, and the risk of tumor‐combined VTE increases sharply during this period. It is possible to measure NETs of tumor patients on the third day after surgery and the day of VTE occurrence, respectively, and compare the difference in NETs between the two groups at these three time points. (iii) The follow‐up time in this study was short, and some tumor patients may develop VTE later. Mauracher et al.^37^ found that tumor patients with elevated citH3 levels had an increased cumulative risk of VTE at 6 months, 1 year, and 2 years, while elevated cfDNA levels were only associated with a higher risk of VTE during the first 3–6 months. Therefore, extending the follow‐up time should allow for more information about the relationship between NETs and thrombosis formation.

NETs can promote the proliferation, invasion, and metastasis of malignant tumors within the TME through various mechanisms. In this study, we divided the patients in the tumor group and each type of tumor group according to clinical stage and found that the levels of citH3 and cfDNA were generally higher in patients with advanced stage tumors. This suggests that elevated levels of NETs in the peripheral blood can reflect tumor progression. The citH3 levels in gliomas^38^ and cfDNA levels in breast cancer,^39^ pancreatic cancer,^40^ and gliomas^38^ have also been found to be associated with clinical staging. In this study, only cfDNA was observed to correlate with the clinical stage of tumors in lung cancer patients, while there was no significant difference in citH3 levels between early‐stage and advanced‐stage patients. In a previous study, measurements of MPO‐DNA complex levels in the peripheral blood of lung cancer patients showed that advanced patients (Stage II–III) had higher levels than those in early patients (Stage I).^41^ Considering the complex composition of NETs and the lack of standardized measurements, there may be some deviation in the levels of their different components. In a recent clinical study on ovarian cancer and NETs, the levels of citH3 and cfDNA in the peripheral blood of ovarian cancer patients were not related to clinical staging.^42^ However, since the inclusion and exclusion criteria for ovarian cancer patients and the specific sampling time of specimens were not mentioned in this study, it was difficult to determine if the influence of other related diseases on NETs could be maximally excluded. Furthermore, previous studies on ovarian cancer and NETs have shown conflicting results,^14^, ^43^, ^44^ indicating a dual impact of NETs on tumors. This suggests that future research on NETs should consider multiple factors.

NETs can promote tumor invasion and metastasis in the TME by inducing tumor‐related inflammatory reactions.^45^ Recent clinical studies on endometrial cancer have shown that citH3 levels in the peripheral blood are positively correlated with lymphocyte counts, while cfDNA levels are positively correlated with monocyte, lymphocyte, and platelet counts, indicating that NETs may be related to lymphocyte‐mediated inflammatory responses.^36^ In this study, we found that cfDNA levels in the peripheral blood of tumor patients were positively correlated with systemic inflammation‐related parameters Ne, M, NLR, MLR, SII, and SIRI, and negatively correlated with Ly. As NETs are associated with neutrophil‐associated and monocyte‐dominated inflammatory responses, these results provide evidence for the involvement of NETs in tumor‐induced systemic inflammation. Furthermore, we found that the association between cfDNA in advanced‐stage tumor patients and the aforementioned systemic inflammation‐related parameters were closer than in early‐stage tumor patients. Additionally, the results of this study showed that the levels of NLR, MLR, PLR, SII, and SIRI were higher in advanced‐stage patients, especially in ovarian and lung cancer patients. The elevation of these five indicators is usually the result of increased neutrophils, monocytes, and platelets, or a decrease in lymphocytes. The abundant recruitment of neutrophils in primary and metastatic tumor sites is related to an increase in granulocyte colony‐stimulating factor expression.^46^ Tumor‐derived interleukin‐6 stimulates hepatic synthesis of thrombopoietin, resulting in increased platelet production.^47^ Moreover, neutrophils, monocytes, and platelets can also promote tumor progression by inhibiting lymphocyte proliferation and function.^48^, ^49^, ^50^, ^51^, ^52^ Therefore, elevation of systemic inflammation‐related parameters NLR, MLR, PLR, SII, and SIRI levels is associated with disease progression in tumor patients.

## 5 CONCLUSION

In summary, the levels of NETs and systemic inflammation‐related parameters in the peripheral blood of tumor patients can increase concomitantly with increased tumor clinical stage. There is also a correlation between these factors, which is more significant in patients with advanced tumors, suggesting that NETs may participate in tumor‐induced systemic inflammatory responses through interaction with circulating inflammatory cells. NETs are involved in the occurrence and development of tumors, but the potential mechanism of their multiple effects on tumors requires further clarification. This study provides new insight for future research into NETs‐based tumor diagnostic tools.

## AUTHOR CONTRIBUTIONS

**Min Wang:** Conceptualization (equal); data curation (equal); formal analysis (equal); investigation (equal); methodology (equal); resources (equal); validation (equal); visualization (equal); writing – original draft (equal); writing – review and editing (equal). **Xiaoyan Lv:** Conceptualization (equal); data curation (equal); investigation (equal); supervision (equal); validation (equal); visualization (equal); writing – review and editing (equal). **Ying Wang:** Formal analysis (equal); investigation (equal); project administration (equal); visualization (equal); writing – original draft (equal). **Yao Li:** Investigation (equal); resources (equal); visualization (equal); writing – original draft (equal). **Honghong Li:** Data curation (equal); formal analysis (equal); investigation (equal); writing – original draft (equal). **Zhongjun Shen:** Investigation (equal); project administration (equal); visualization (equal); writing – original draft (equal). **Liyan Zhao:** Conceptualization (equal); methodology (equal); project administration (equal); supervision (equal); validation (equal); writing – review and editing (equal).

## FUNDING INFORMATION

This study was funded by the Jilin Province Science and Technology Development Planning Project (20200801023GH).

## CONFLICT OF INTEREST STATEMENT

The authors declare that the research was conducted in the absence of any commercial or financial relationships that could be construed as a potential conflict of interest.

## Supporting information


Figure S1:

## Data Availability

All datasets generated for this study are included in the article/supplementary material.
