# Supplementary figures and images for: Biomarkers of peripheral blood neutrophil extracellular traps in the diagnosis and progression of malignant tumors
Source: Cancer Med. 2024 Jan 17;13(3):e6935. doi: 10.1002/cam4.6935 (PMC10905219; doi:10.1002/cam4.6935)

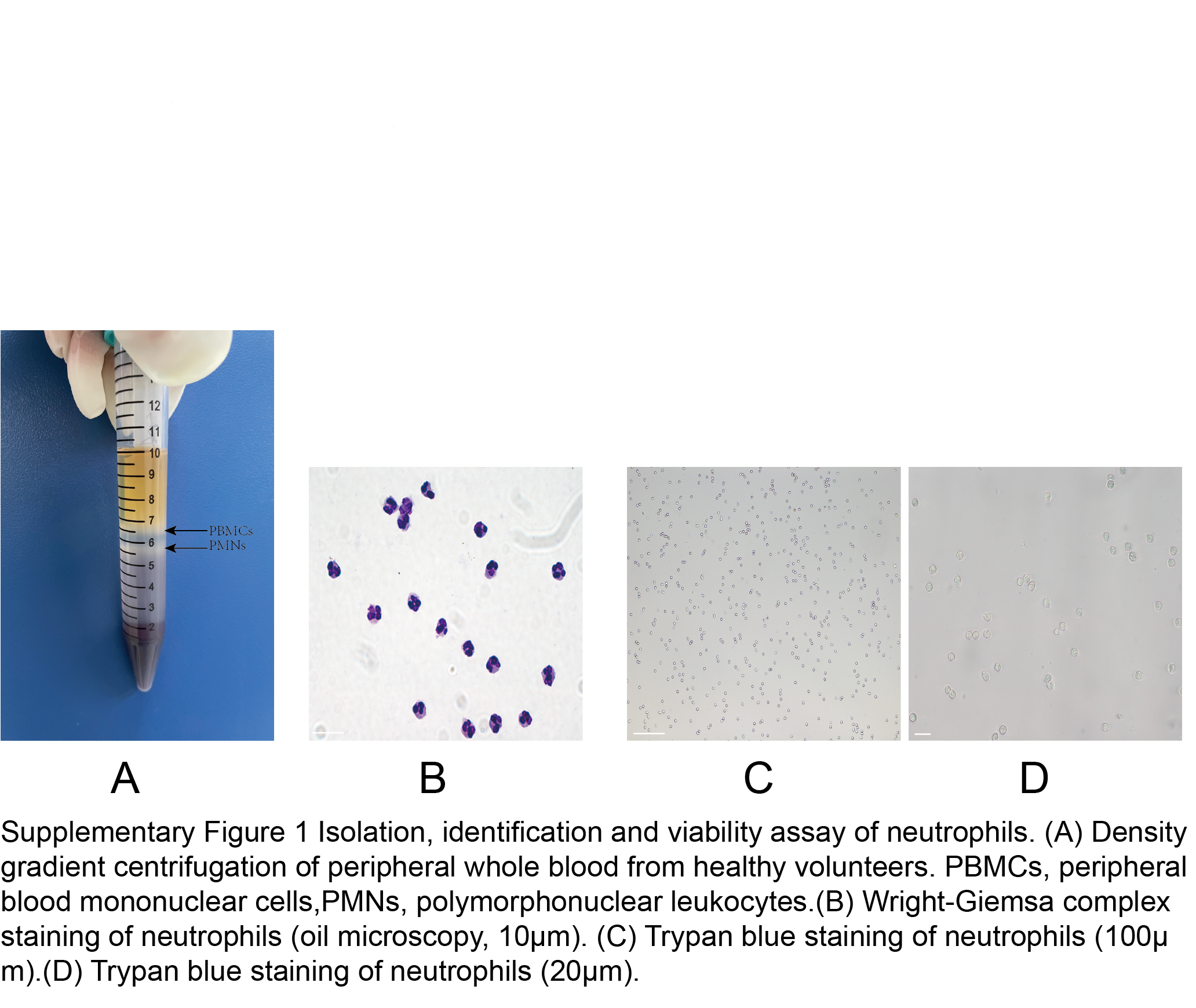

Supplement: Supplementary file 1 — Figure S1: [file CAM4-13-e6935-s001.tif]
